# Arctiin Inhibits Cervical Cancer Cell Migration and Invasion through Suppression of S100A4 Expression via PI3K/Akt Pathway

**DOI:** 10.3390/pharmaceutics14020365

**Published:** 2022-02-05

**Authors:** Chung-Yuan Lee, Min-Chieh Hsin, Pei-Ni Chen, Chiao-Wen Lin, Po-Hui Wang, Shun-Fa Yang, Yi-Hsuan Hsiao

**Affiliations:** 1 Department of Obstetrics and Gynecology, Chiayi Chang Gung Memorial Hospital, Chiayi 613, Taiwan; littlemice6@gmail.com; 2 Department of Nursing, Chang Gung University of Science and Technology, Chiayi Campus, Chiayi 613, Taiwan; 3 Institute of Medicine, Chung Shan Medical University, Taichung 402, Taiwan; sinmusha@hotmail.com (M.-C.H.); peini@csmu.edu.tw (P.-N.C.); wang0860@csmu.edu.tw (P.-H.W.); 4 Department of Medical Research, Chung Shan Medical University Hospital, Taichung 402, Taiwan; 5 Institute of Oral Sciences, Chung Shan Medical University, Taichung 402, Taiwan; cwlin@csmu.edu.tw; 6 Department of Dentistry, Chung Shan Medical University Hospital, Taichung 402, Taiwan; 7 Department of Obstetrics and Gynecology, Chung Shan Medical University Hospital, Taichung 402, Taiwan; 8 School of Medicine, Chung Shan Medical University, Taichung 402, Taiwan; 9 Department of Obstetrics and Gynecology, Changhua Christian Hospital, Changhua 500, Taiwan; 10 Women’s Health Research Laboratory, Changhua Christian Hospital, Changhua 500, Taiwan

**Keywords:** arctiin, S100A4, migration, cervical cancer

## Abstract

Arctiin, a lignan glycoside, is isolated from *Arctium lappa* L. The anticancer effects of arctiin have been demonstrated in several studies. However, no research has been conducted on the anti-migration effect of arctiin in cervical cancer cells. The present study examined the effects of arctiin on cervical cancer cells and investigated the possible molecular mechanism. We demonstrated that arctiin exhibited low cytotoxicity and significantly inhibited cell migration and invasion in human cervical cancer cells. The S100A4 protein expression and mRNA levels were significantly reduced in HeLa and SiHa cells with arctiin treatment. Furthermore, silencing S100A4 by using small interfering RNA reduced cell migration, while overexpression of S100A4 mitigated the migration inhibition imposed by arctiin in cervical cancer cells. Western blotting revealed that arctiin significantly reduced phosphoinositide 3-kinase (PI3K) and phosphorylation of Akt in cervical cancer cells. Moreover, selective Akt induction by an Akt activator, SC-79, reverted cervical cancer cell migration and S100A4 protein expression, which were reduced in response to arctiin. Taken together, these results suggest that arctiin inhibits cervical cancer cell migration and invasion through suppression of S100A4 and the PI3K/Akt pathway.

## 1. Introduction

As the fourth most common malignancy diagnosed in women worldwide, cervical cancer is a global health concern [1]. Notably, patients with cervical cancer with metastasis have poor prognosis, with a median survival of 8–13 months [2]. The 5-year survival rate is 16.5% for metastatic cervical cancer, compared with 91.5% for localized cervical cancer [3,4]. Treatment options for patients with early-stage cervical cancer and locally advanced cervical cancer include surgery, chemotherapy, or radiotherapy. However, no standard treatment is available for patients with metastatic cervical cancer because of its heterogeneous manifestations [3]. Because treatment options for metastatic disease are limited, palliative treatment is the major consideration [2]. Therefore, enhancing the survival of patients with metastatic cervical cancer through new therapeutic agents or treatment strategies is imperative.

S100A4, a member of the S100 family of calcium-binding proteins, plays a key role in the process of human tumorigenesis and metastasis [5]. S100A4 exhibits an increased level in various types of cancer [5]. Moreover, S100A4 is involved in various physiological functions, such as cell migration, invasion, and adhesion [6,7,8,9]. Overexpression of S100A4 was observed in several types of metastatic cancer, including epithelial ovarian carcinoma [10], prostate cancer [11], breast cancer [12], and pancreatic cancer [13]. S100A4 promotes lung tumor development; a high expression of S100A4 in lung adenocarcinoma tissue is associated with poor prognosis [14]. Furthermore, Liu et al., reported that elevated S100A4 expression can be regarded as an unfavorable prognostic indicator in patients with cervical cancer [15]. However, the role of S100A4 in the migration and invasion of cervical cancer is unclear.

Arctiin, a lignan glycoside, is isolated from *Arctium lappa* L. The anti-inflammatory effects of arctiin have been widely investigated [16]; its anticancer effects were demonstrated in human multiple myeloma cells [17]. Moreover, the bioactivity of arctiin was evidenced by significant induction of cell detachment in prostate cancer PC-3 cells [18]; its cell growth inhibition effect was observed in human immortalized keratinocyte HaCaT cells [19]. Several studies have revealed the anticancer effect of arctiin; however, no study has investigated the effects of arctiin on cervical cancer cells. Hence, this study examined the effects of arctiin, with a potential anti-migration effect, on human cervical cancer HeLa and SiHa cells in vitro to determine the molecular pathway.

## 2. Materials and Methods

### 2.1. Cell Lines and Culture

Human cervical cancer cell lines: HeLa and SiHa were cultured in Dulbecco’s modified Eagle’s medium (DMEM). Human proximal tubular (PTC) cell line HK-2 were cultured in Dulbecco’s Modified Eagle’s Medium (DMEM)/Ham’s F-12 mix medium. We supplemented DMEM with 10% fetal bovine serum (FBS) and 100 ng/mL each of penicillin and streptomycin (P/S). Both of HeLa and SiHa cervical cancer cells were cultured at 37 °C in a humidified atmosphere of 5% CO_2_. Authentication of cancer cell lines by short tandem repeat (STR) DNA profiling analysis was performed (Genomics Co., https://en.genomics.com.tw/, accessed on 5 January 2022). The workflow included DNA extraction, multiplex PCR, fluorescent capillary electrophoresis, data analysis with GeneMapper v4.0 and STR profiling analysis with the DSMZ database. The results were highly similar to SiHa (DSMZ Number: HTB-35) (Evaluation value (EV) value: 0.94) and HeLa.P3 (DSMZ Number: JCRB0649.1) (EV value: 0.89) based on comparison with the DSMZ database.

### 2.2. Cell Cytotoxicity

Arctiin was bought form Sigma-Aldrich (CAS Number: 20362-31-6). HeLa, SiHa and HK-2 cells were seeded in 24-well plates and stimulated with different concentrations of Arctiin (0, 10, 20, 40, 80 μM) for 24 h. After that, we removed the medium and carefully washed it with 1X PBS. MTT (Sigma) were added to each well and incubated at 37 °C in 5% CO_2_ for 4 h. The viable cells were detected spectrophotometrically at 563 nm (Beckman Spectrophotometer DU640; Beckman Instruments, Fullerton, CA, USA).

### 2.3. Migration and Invasion Assay

HeLa and SiHa cells were treated with different concentrations of Arctiin (0, 10, 20, 40 μM) or SC-79 (10 μM) for 24 h. Then, we collected the cells by Trypsin (Gibco) and the cell migration assay in vitro was seeded in a Boyden chamber (Neuro Probe). For the migration assay: Treated cells in 0.5% FBS medium were loaded into the well of the chamber at the upper part and incubated at 37 °C in 5% CO_2_ for 24 h. For invasion: 10 μL Matrigel (BD Biosciences, MA, USA) was applied to 8 μm pore size polycarbonate membrane filters that included 10% FBS DMEM medium and then air-dried for 3~4 h in an incubator. The migrated and invaded cells were fixed by methanol for 10 min, stained with Giemsa and counted by light microscopy [20].

### 2.4. CS2-Empty Vector and CS2-S100A4 Transfection

HeLa and SiHa cells were seeded into 6-cm plates and cultured for 16~18 h. After that we transfected 5 µg of the empty CS2-vector (GenDiscovery Biotechnology, Taipei, Taiwan) or CS2-S100A4 (a gift from Dr. Isao Matsuura, National Health Research Institutes, Taiwan) into the cells and left for 6 h before carefully removing the reagent and culturing the cells with fresh 10% FBS DMEM medium overnight. The next day, the transfected cells were treated with Arctiin (0 and 40 μM) for another 24 h and analysis was performed by the migration assay and the Western blot assay.

### 2.5. Western Blot Assay

The cell lysates which were treated with Arctiin (0, 10, 20, 40 μM) or SC-79 (10 μM) that separated in a polyacrylamide gel and transferred onto PVDF membranes. Membranes were incubated with 5% non-fat milk in TBST for 1 h in room temperature and, subsequently, covered with a corresponding antibody against a specific protein overnight at 4 °C. Next, we removed the primary antibody and washed it with TBST buffer for 7 min, three times. Signal was detected by the ECL solution system. Antibodies used were as follows: anti-S100A4 (#ab124805, Abcam), anti-p-JNK (#4668, Cell Signaling), anti- JNK (#9258, Cell Signaling), anti-p-ERK (#4370, Cell Signaling), anti-ERK (#9102, Cell Signaling), anti-p-P38 (#4511, Cell Signaling), anti-P38 (#612168, BD), anti-p-Akt (#4060, Cell Signaling), anti-Akt (#610860, BD), anti-PI3K (#610045, BD) and anti-β-actin (#ab8226, Abcam).

### 2.6. Real-Time RT-PCR

HeLa and SiHa cells were treated with Arctiin (0, 10, 20, 40 μM) or SC-79 for 24 h, and total RNA was extracted using the TRIzol reagent. For reverse transcription, the first-strand cDNA was generated using the high-capacity cDNA reverse transcription kit. PCR amplification was performed as described previously [21]. S100A4 gene primers were designed by Primer3 software. S100A4 gene expression was measured using the comparative Ct method.

### 2.7. Statistical Analyses

Statistically significant differences were calculated using the Student’s *t*-test (SigmaPlot 10.0, Jandel Scientific, and San Rafael, CA, USA). Significance was set at *p* < 0.05. The values are the means ± standard deviation (SD) of at least three independent experiments.

## 3. Results

### 3.1. Effects of Arctiin on the Viability of Cervical Cancer Cells

**Figure 1:**
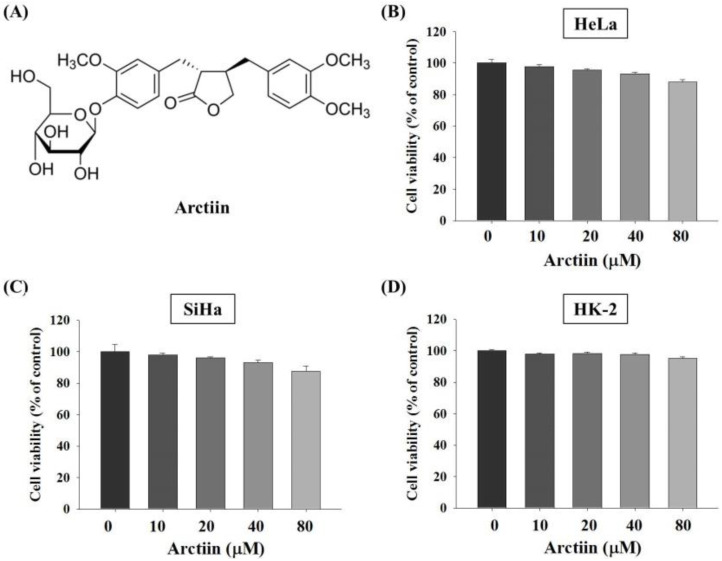
Effects of arctiin on the cell viability of HeLa, SiHa and human proximal tubular HK-2 cells. (**A**) Chemical structure of arctiin. (**B**) HeLa, (**C**) SiHa and (**D**) HK-2 cells were treated with various concentrations (0, 10, 20, 40 and 80 μM) of arctiin for 24 h before being subjected to an MTT assay for cell viability. The values represented the means ± SD of at least 3 independent experiments.

The effects of arctiin (Figure 1A) at various concentrations (0, 10, 20, 40, and 80 μM) on HeLa, SiHa and human proximal tubular HK-2 cells are indicated in Figure 1. Results of the MTT assay revealed no significant differences in cancer cell viability between cells in different concentrations (Figure 1B–D). These results indicated that arctiin did not exert significant cytotoxic effects in cell viability. Therefore, arctiin concentrations of 0–40 μM were used in all subsequent experiments.

### 3.2. Inhibitory Effects of Arctiin on Cell Migration and Invasion in Cervical Cancer Cells

**Figure 2:**
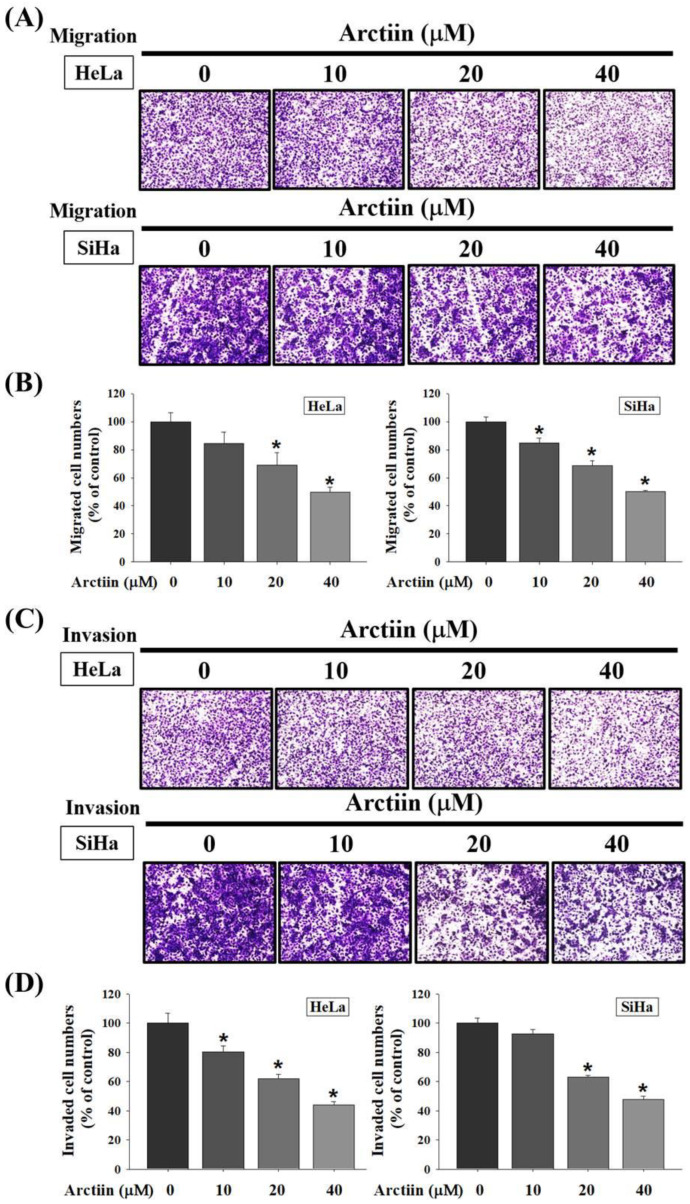
Effects of arctiin on cell migration and invasion of HeLa and SiHa cells. After being treated with arctiin at a concentration of 0, 10, 20, and 40 μM for 24 h, the cell migration and invasion were measured using a Boyden chamber. The number of cells that invaded the underside of the porous polycarbonate was accounted for by assessing the migration (**A**,**B**) and invasion (**C**,**D**) abilities of HeLa and SiHa cells. The values represented the means ± SD of at least 3 independent experiments. * *p* < 0.05, compared with the group that was without arctiin treatment.

Cell migration and invasion assays with the Boyden chamber were used to determine the effects of arctiin on HeLa and SiHa cells. For cells treated with various arctiin concentrations (0–40 μM), the cell migration abilities of HeLa were significantly reduced by arctiin compared with the control group (Figure 2A,B). In addition, the cell migration abilities of SiHa were significantly reduced by arctiin (Figure 2A,B). Similarly, for cells treated with different arctiin concentrations (0–40 μM), the cell invasion abilities of HeLa and SiHa cells were significantly reduced by arctiin compared with the control (Figure 2C,D). These results suggested that arctiin significantly reduced the cell migration and invasion of cervical cancer cells in a concentration-dependent manner.

### 3.3. Effects of Arctiin on the S100A4 Protein and Mrna Level

**Figure 3:**
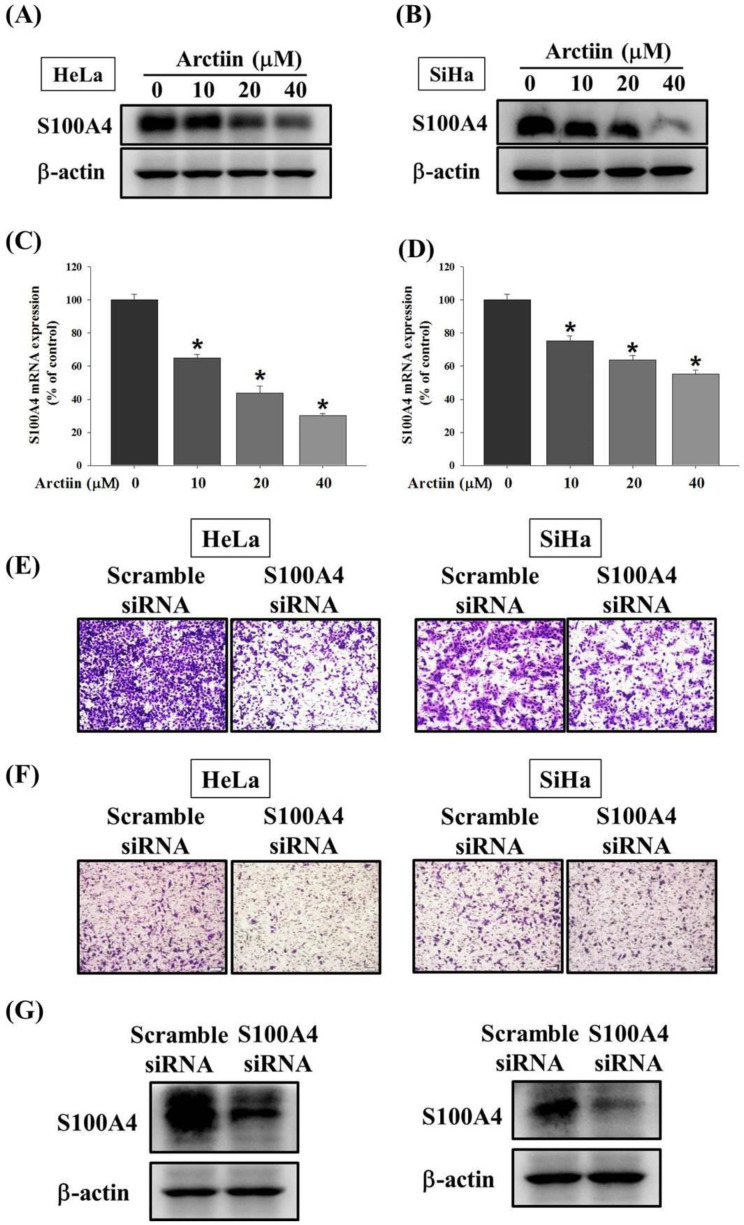
Effects of arctiin on the S100A4 protein expression and mRNA levels of HeLa and SiHa cells. After being treated with arctiin at a concentration of 0, 10, 20, and 40 μM for 24 h, the S100A4 protein and mRNA expression of HeLa (**A**,**C**) and SiHa (**B**,**D**) were measured by Western blotting assay and real time PCR assay, respectively. After silencing S100A4 in the HeLa cells and SiHa cells, (**E**) Boyden chamber assay, (**F**) Boyden chamber invasion assay, and (**G**) Western blot were used for analysis. The values represented the means ± SD of at least 3 independent experiments. * *p* < 0.05, compared with the group that was without arctiin treatment.

S100A4 protein and messenger ribonucleic acid (mRNA) expression were significantly reduced in HeLa cells treated with arctiin (10, 20, and 40 μM) compared with the control (Figure 3A,C). Similarly, arctiin reduced S100A4 protein and mRNA level in the SiHa cells (Figure 3B,D). Moreover, the transfection of S100A4-specific small interfering RNA significantly reduced S100A4 protein expression, concomitantly lowering the migratory and invasion ability in the HeLa and SiHa cancer cells (Figure 3E–G).

**Figure 4:**
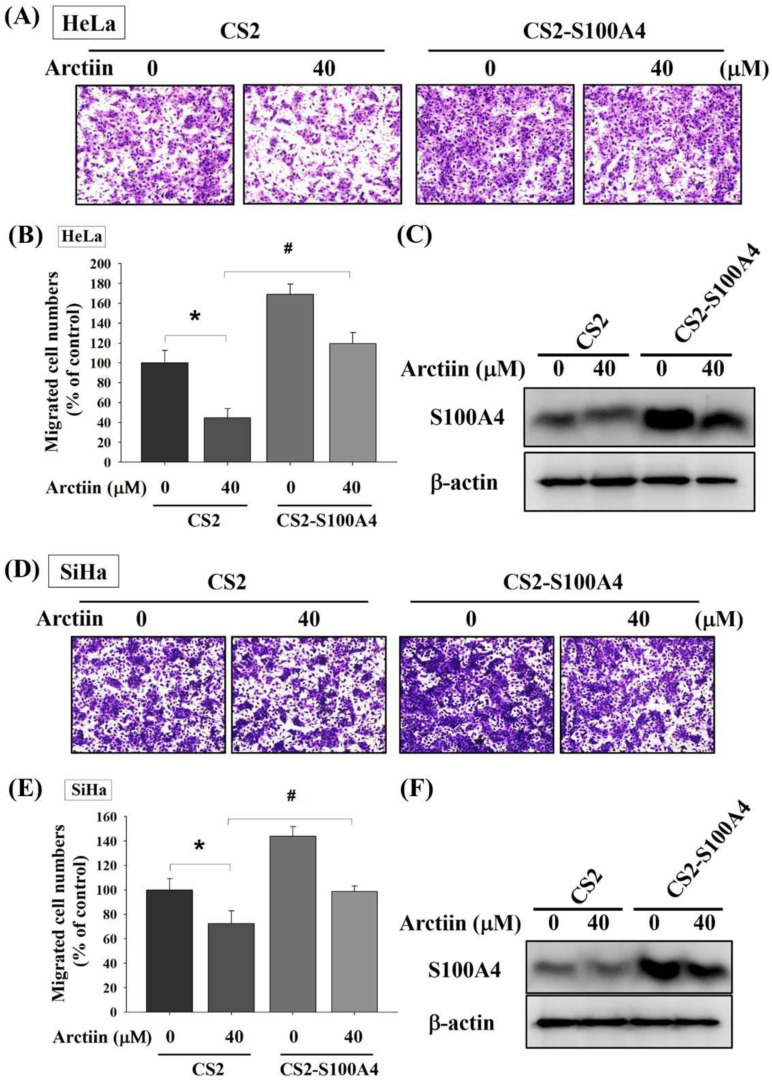
Effects of S100A4 on cell migration and invasion of HeLa and SiHa cells. (**A**–**C**) Cell migration assays and Western blot analyses for HeLa cells with or without arctiin treatment or overexpression of S100A4 (CS2-S100A4), were measured, respectively. (**D**–**F**) Cell migration assays and Western blot analyses for SiHa cells with or without arctiin treatment or overexpression of S100A4, were measured, respectively, and migratory cells were subsequently subjected to quantitative analysis. * *p* < 0.05, compared with the control group. # *p* < 0.05, comparison with the arctiin-treated group.

To determine the effect of S100A4 expression on cervical cancer cell migration, the overexpression vector of S100A4 (CS2-S100A4) was used. Boyden chamber assay and Western blotting revealed that S100A4 overexpression markedly increased cell migration activity and S100A4 protein expression in HeLa cells, respectively (Figure 4A–C). We combined the overexpression of S100A4 with arctiin treatment, which revealed that S100A4 overexpression mitigated the expression of S100A4 protein and inhibition of cell migration imposed by arctiin in HeLa cells. Similar results were observed in SiHa cancer cells (Figure 4D–F).

### 3.4. Effects of Arctiin on PI3K/Akt Pathway in Cervical Cancer Cells

**Figure 5:**
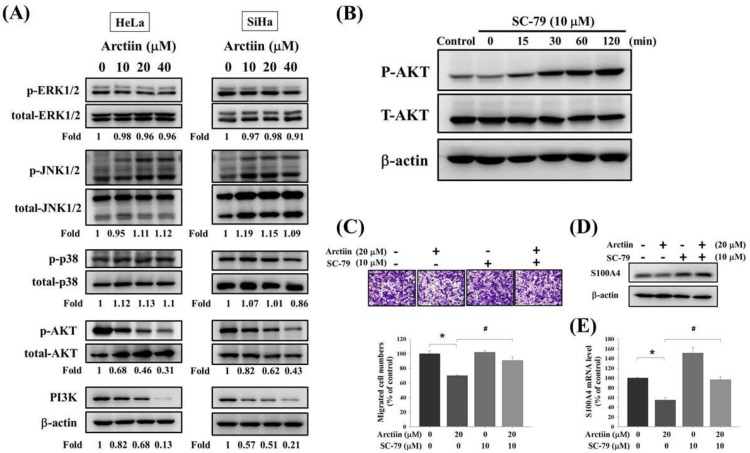
Effects of arctiin on PI3K/Akt pathway in HeLa and SiHa cells. (**A**)**.** After being treated with arctiin at a concentration of 0, 10, 20 and 40 μM for 24 h, the phosphorylation of ERK1/2, JNK1/2, p38, AKT and PI3K for HeLa and SiHa were analyzed by Western blotting assay. (**B**). After being treated with Akt activator SC-79 for 0, 15, 30, 60 and 120 min, the phosphorylation of AKT for SiHa were analyzed by Western blotting assay. After being treated with 20 μM arctiin in the presence or absence of SC-79 (10 μM) for 24 h, (**C**) the migrated cell number, (**D**) S100A4 protein expression and (**E**) S100A4 mRNA levels were analyzed. * *p* < 0.05, compared with the control group. # *p* < 0.05, comparison with the arctiin-treated group.

To further analyze the mechanisms involved in arctiin-mediated S100A4 suppression, Western blotting was used to evaluate the effects of arctiin on the expression of the mitogen-activated protein kinase (MAPK) and phosphoinositide 3-kinase (PI3K)/Akt pathways. Western blotting demonstrated that arctiin significantly reduced PI3K expression and phosphorylation of Akt in HeLa and SiHa cells in a concentration-dependent manner (Figure 5A). However, the phosphorylation of extracellular signal-regulated protein kinase 1/2 (ERK1/2), c-Jun N-terminal kinase 1/2 (JNK1/2) and p38 was not altered by arctiin treatment (Figure 5A). Moreover, to confirm the effect of the PI3K/Akt pathway on arctiin-treated SiHa cell line, the Akt activator SC-79 was used. As shown in Figure 5B, Western blotting demonstrated that SC-79 significantly increased phosphorylation of Akt in SiHa cells (Figure 5B). Furthermore, Boyden chamber assay, Western blotting and RT-PCR assay revealed that the combination of SC-79 and arctiin could reverse the arctiin-mediated suppression of the migratory ability, S100A4 protein expression and S100A4 mRNA level in SiHa cells, respectively (Figure 5C–E).

## 4. Discussion

Various Chinese herbal medicines or herbal products have been investigated for the treatment of cervical cancer [22]; the anticancer effects of arctiin have been demonstrated in several studies. In prostate cancer PC-3 cells, arctiin induced cell detachment and lowered cell numbers through modulation of antiadhesion molecule MUC-1 [18]. The antiproliferative effect of arctiin on several types of human cancer cells, including osteosarcoma, colorectal cancer, melanoma, lung cancer, breast cancer, prostate cancer, and transformed renal cells, was reported to involve the downregulation of cyclin D1 expression [19]. Moreover, the antihepatocarcinogenic effects of arctiin on male F344 rats was revealed through the inhibition of the glutathione S-transferase [23]. The antitumor-promoting effect of arctiin was demonstrated through carcinogenesis testing of mouse pulmonary tumors [24]. Our study revealed the anti-migration effects of arctiin on cervical cancer through the inhibition of S100A4 expression and the PI3K/Akt pathway.

The S100A4 protein, a member of the S100 family, has been discovered to have a potent role in inflammation-associated diseases and the biological functions of cell differentiation, angiogenesis, apoptosis, migration, and invasion [25]. Increased expression levels of S100A4 were observed in various types of cancers; thus, S100A4 is a key driver of tumorigenesis and metastasis [5]. S100A4 could be used as a promising molecule for target therapies or as a metastasis biomarker [5]. Boye et al., reported that S100A4′s promotion of the metastatic capacity of cancer cells could be the result of the turnover of myosin IIA filaments of migrating cells [7]. Fei et al., demonstrated the regulation of invasion and metastasis by S100A4 in colorectal cancer cells [26]. Hepatocellular carcinogenesis promoted by A100A4 was also reported [27]. S100A4 acted synergically with the extracellular matrix in the progression of hepatocellular carcinoma by affecting the stemness of cancer cells [27]. In our study, the expressions of S100A4 mRNA and protein were significantly reduced in cervical cancer cells treated with arctiin compared with the control in a concentration-dependent manner.

The PI3K/Akt pathway regulates a broad spectrum of cellular processes, including cell growth, proliferation, metabolism, motility, survival, and apoptosis [28]. Alterations to the PI3K/Akt signaling pathway are frequently observed in cancer; therefore, specific inhibition of the activation of Akt may be a reasonable promising approach for treating cancer [29,30]. The effect of the PI3K/Akt signaling pathway on bone metastasis of prostate carcinoma has been investigated [31]. The PI3K/Akt signaling pathway activates the expressions of receptor activator of nuclear factor kappa-B ligand, parathyroid hormone related protein, and bone morphogenetic protein-2 partly through nuclear factor kappa B (NF-κB); hence, it plays a crucial role in the process of prostate cancer metastasis to bone [31].

Several compounds exert anticancer effects on cervical cancer through Akt signaling pathways [32]. Chelidonine isolated from the ethanolic extract of *Chelidonium majus* resulted in the induction of apoptosis in HeLa cells through possible alterations of the PI3K/Akt and p38 signaling pathways [32]. Butein, a bioactive flavonoid isolated from numerous native plants, reduced the phosphorylation of PI3K, Akt, and mammalian target of rapamycin (mTOR) expression and contributed to the inhibition of the tumor growth of HeLa cervical cancer cells [33]. RCE-4, the main active composition of *Reineckia carnea*, exerted an anti-cervical cancer effect on HeLa cells by reducing PI3K, Akt, mTOR, and NF-κB p65 phosphorylation levels [34]. In a study of colorectal cancer, adenovirus-mediated S100A4 overexpression enhanced the viability and migration of colorectal cancer cells; these effects of S100A4 were reduced by treatment with the specific PI3K/Akt signaling inhibitor LY294002 [35]. A breast cancer model study revealed that metastatic tumors exhibited high S100A4 expression and contained a high percentage of epithelial-mesenchymal transition signature-positive cells [36]. Because S100A4 is involved in metastasis in this model of PIK3CA/TP53 double-positive cancers, S100A4 could be a diagnostic and therapeutic target [36]. In our study, treatment with arctiin inhibited cervical cancer cell migration and invasion through the suppression of the PI3K/Akt pathway. These results suggest that the PI3K/Akt pathway has a crucial role in mediating cervical cancer cell migration.

## 5. Conclusions

In conclusion, our study revealed that arctiin inhibits cervical cancer cell migration and invasion through the suppression of S100A4 expression and the PI3K/Akt pathway. However, our study was limited by the lack of an in vivo animal study. Therefore, further pharmacological and clinical studies are required to verify the therapeutic potential of arctiin in cervical cancer.

## Data Availability

The data used to support the findings of the present study are available from the corresponding author upon request.
